# Reducing Radiation Dose in Computed Tomography Imaging of Adolescent Idiopathic Scoliosis Using Spectral Shaping Technique with Tin Filter

**DOI:** 10.3390/tomography11100110

**Published:** 2025-09-29

**Authors:** Yoshiyuki Noto, Tatsuya Kuramoto, Kei Watanabe, Koichi Chida

**Affiliations:** 1 Division of Radiological Technology, Department of Technical Support, Niigata University Medical and Dental Hospital, Niigata 951-8520, Japan; yoshino@med.niigata-u.ac.jp (Y.N.); tkuramoto@med.niigata-u.ac.jp (T.K.); 2 Course of Radiological Technology, Health Sciences, Tohoku University Graduate School of Medicine, Sendai 980-8575, Japan; 3 Division of Orthopedic Surgery, Department of Regenerative and Transplant Medicine, Niigata University Graduate School of Medical and Dental Sciences, Chuo Ward, Niigata 951-8520, Japan; keiwatanabe_39jp@live.jp; 4 Department of Radiation Disaster Medicine, International Research Institute of Disaster Science, Tohoku University, Sendai 980-0845, Japan

**Keywords:** adolescent idiopathic scoliosis, spectral shaping technique, computed tomography (CT), radiation dose, radiation reduction, tin filter, low-dose CT, radiation-induced cancers, children, radiosensitivity

## Abstract

**Background/Objectives**: Children with adolescent idiopathic scoliosis (AIS) require repeated imaging, primarily standing spine radiography, while CT may be required for surgical planning, resulting in higher radiation exposure. Spectral shaping using a tin filter can reduce radiation dose in non-contrast chest CT. This study evaluated the efficacy of spectral shaping using a tin filter for reducing radiation dose in CT imaging in AIS and its impact on image quality. **Methods**: We retrospectively analyzed 51 AIS patients who underwent spine CT between February 2017 and March 2022, and divided them into two groups: normal-dose CT (NDCT) and low-dose CT with spectral shaping with a tin filter (LDCT). Radiation doses and image quality were compared between the groups. Radiation dose was recorded as the volume CT dose index (CTDIvol) and the dose length product emitted from the device, and effective and equivalent doses obtained from simulations. **Results**: The use of spectral shaping with a tin filter resulted in a 75% reduction in radiation dose compared to conventional CT without any reduction in image quality. **Conclusions**: Spectral shaping CT with a tin filter can substantially reduce radiation dose while maintaining image quality. It may be considered a safer alternative to conventional CT when clinically indicated in AIS patients.

## 1. Introduction

Radiation exposure from medical imaging has become an increasingly important issue in pediatric imaging [1,2]. While radiological examinations are essential for accurate diagnosis and treatment planning [3], they also carry a potential risk of radiation-induced malignancies, particularly in children who are more radiosensitive than adults. The balance between clinical benefit and radiation risk must be carefully considered [4,5,6], especially in conditions requiring repeated imaging such as scoliosis. In this context, dose reduction strategies are of paramount importance to minimize long-term health risks [7,8,9,10].

**Figure 1:**
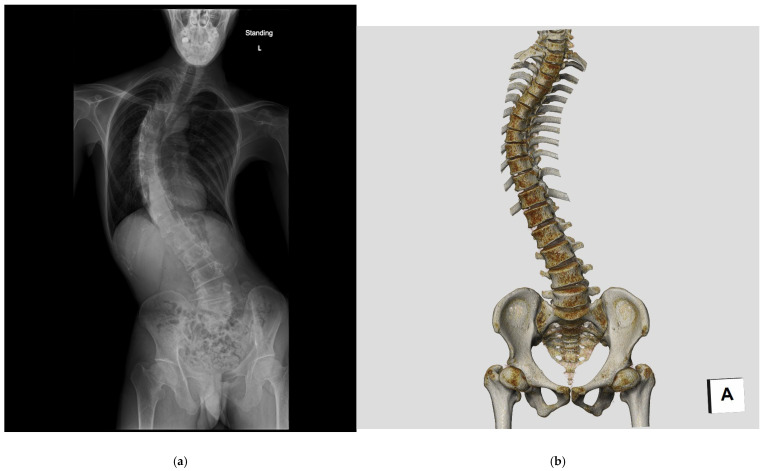
Radiographic and CT images of a patient with adolescent idiopathic scoliosis (AIS). (**a**) Standing postero-anterior (PA) radiograph of the whole spine. (**b**) Three-dimensional computed tomography (3DCT) image reconstructed using volume rendering.

Scoliosis is a three-dimensional deformity of the spine that develops due to various etiological factors. (Figure 1). Adolescent idiopathic scoliosis (AIS), the most prevalent type of scoliosis, accounting for 80–90% of all cases, occurs during the growth period, particularly in the upper elementary to junior high school grades. The incidence of AIS is reportedly five to seven times higher in females than males, with a >90% incidence rate in identical twins [11]. Gradually, genetic predisposition to AIS is increasingly being studied; studies have pointed out a positive correlation between maternal scoliosis and the probability of developing scoliosis in children [12,13].

Precise and reliable evaluation of AIS requires the use of radiation, i.e., serial X-ray examinations are mandatory for both diagnosis and follow-up of AIS [14]; however, this is accompanied by a risk of radiation-induced carcinogenesis [15,16,17]. Radiation provides valuable information, but also involves exposure.

Recent advances in diagnostic imaging have led to the introduction of 3D ultrasound as a useful tool for the assessment of scoliosis. Studies have demonstrated its usefulness in postural analysis and Cobb angle measurement, making it a non-invasive alternative [18,19]. However, these techniques are not yet widely available and remain as adjuncts to assessment using radiographs and computed tomography (CT) scans.

The standard examination for AIS is standing full-spine radiography, with an effective dose of approximately 0.05 mSv for a single projection (PA). As the standard examination consists of both PA and lateral projections, the cumulative effective dose is approximately 0.1 mSv. [20]. In certain clinical situations, such as surgical planning and postoperative evaluation, CT scans are also performed, but the dose is significantly higher, around 8 mSv [21]. Therefore, justification and optimization are essential [22]. Childhood is a period of heightened radiosensitivity, and patients with scoliosis are known to have an elevated risk of breast cancer, leukemia, and uterine cancer [14,23,24]. At present, corrective AIS surgery necessitates CT imaging for safety and to assess the effectiveness of surgical interventions using navigation systems [25]. Although the radiation dose associated with CT is higher than that with plain radiography [26], tube current modulation mechanisms, such as auto-exposure control (AEC) and iterative reconstruction, which are employed to facilitate a reduction in image noise, allow significant cutback of radiation exposure [27,28,29,30,31]. CT with spectrum shaping technology applies additive filter technology from general radiography to CT. It is an upcoming technique that uses a movable tin filter to selectively target non-contrast scans and high contrast regions to significantly reduce radiation exposure. This is achieved by effectively removing the low-energy component of the continuous spectrum and biasing the average energy toward the higher end [32]. However, this technology has only been introduced in certain CT scanners and is not yet available in general CT scanners. Recent investigations have indicated that the radiation dose can be reduced by ~73% in a sinus CT and ~90% in non-contrast chest CTs using spectral shaping technology while retaining the image quality [33,34]. Several studies have reported the usefulness of low-dose CT for AIS patients [35,36]. However, no studies have examined the image quality of low-dose CT for AIS patients using CT with spectrum shaping technology. In this study, we evaluated the efficacy of spectral shaping using a tin filter in reducing radiation exposure in AIS patients by comparing the radiation dose and image quality of low-dose CT (LDCT) using spectral shaping with a tin filter versus those obtained using normal-dose CT conditions (NDCT).

The purpose of this study was to evaluate the efficacy of spectral shaping CT with a tin filter in reducing radiation exposure while maintaining diagnostic image quality in adolescent idiopathic scoliosis patients.

## 2. Materials and Methods

### 2.1. Patient Selection and Study Design

This retrospective study was conducted at a single center and approved by the local ethics committee; as the study was retrospective and the data were anonymized, the Institutional Review Board waived the need for written informed consent.

The present study was approved by the Institutional Review Board of Niigata University Medical and Dental Hospital (2019-0139; exact date of ethical approval, 2 August 2019).

Patients diagnosed with AIS who had undergone spine CT for scoliosis evaluation (n = 51) between 1 February 2017 and 31 March 2022 were included in the study. The inclusion criteria were as follows: (1) confirmed diagnosis of adolescent idiopathic scoliosis, (2) female patients aged 12–19 years, which corresponds to the typical age range during which AIS is diagnosed and surgically treated, and (3) spinal CT performed for preoperative surgical planning. The exclusion criteria were as follows: (1) congenital or neuromuscular scoliosis, and (2) male patients. A total of 23 female patients (median age = 16 years; range: 12–19 years) were included in the LDCT group and 28 female patients (median age = 15 years; range: 13–19 years) in the NDCT group. Patients were allocated to either the LDCT or NDCT group depending on the scanner protocol applied at the time of imaging, which was determined by scheduling and equipment availability rather than intentional randomization.

### 2.2. CT Examination

For the CT examination, the patients were placed in a supine position with their arms elevated and asked to hold their breath in inspiration. The imaging range was from the seventh cervical vertebra to the lower end of the pelvic bone. LDCT was performed using a third-generation dual source CT scanner (DSCT) (Siemens SOMATOM Force, Siemens Healthcare Sector, Forchheim, Germany) using the following scanning parameters: tube voltage = 100 kVp, reference tube current = 300 mAs (100–380 mAs), rotation time = 0.5 s, pitch = 1.0, and detector collimation = 96 raw × 0.6 mm. This protocol included the use of a dedicated 0.6 mm tin filter placed immediately after the X-ray source. All patients underwent automatic tube current modulation using AEC (CareDose 4D, Siemens Healthcare Sector, Forchheim, Germany) to optimize the effective current (in mAs).

NDCT was performed using a Single Source CT scanner (SSCT) (Philips Ingenuity Elite, Royal Philips, Eindhoven, The Netherlands) with the following scanning parameters: tube voltage = 120 kVp, rotation time = 0.4 s, pitch = 1.0, and detector collimation = 64 raw × 0.625 mm. The tube current was set to DRI (Dose Right Index; Royal Philips, Eindhoven, The Netherlands) at 20 mAs (range: 15–70 mAs), and all patients underwent AEC.

### 2.3. Image Reconstruction

The following standard image reconstruction parameters for the spine surgical navigation system were used: reconstructed slice thickness = 1.0 mm, display field of view = 150 mm, and matrix size = 512 × 512.

For the LDCT image reconstruction, the Bf44 kernel and a novel iterative reconstruction technique, the Adaptive Model-based Iterative Reconstruction (Siemens Healthcare, Forchheim, Germany), were used with level three intensity applied for strong noise reduction (intensity range from 1 to 5) [37].

Similarly, for the NDCT image reconstruction, the type C kernel and another iterative reconstruction algorithm called the iDose4 (Royal Philips, Eindhoven, The Netherlands) were used; level four intensity was applied for strong noise reduction (intensity range from one to seven).

### 2.4. Radiation Dose Assessment

The volume CT dose index (CTDIvol) and the dose length product (DLP) for each scanner protocol were recorded for each patient. The effective dose was calculated using the Radimetrics software for dose management (version 2.9, Bayer HealthCare, Whippany, NJ, USA) as per the International Commission on Radiological Protection (ICRP) Publication 103. This software estimates patient dose using a Monte Carlo simulation–based algorithm [38]. The equivalent doses of the mammary gland, uterus, and ovary were also recorded. An analysis was conducted to examine the correlations between effective dose, equivalent dose of the mammary gland, and body indices (height, weight, and body mass index [BMI]), as well as the correlations between CTDIvol, DLP, and these body indices.

### 2.5. Image Quality Assessment

**Figure 2:**
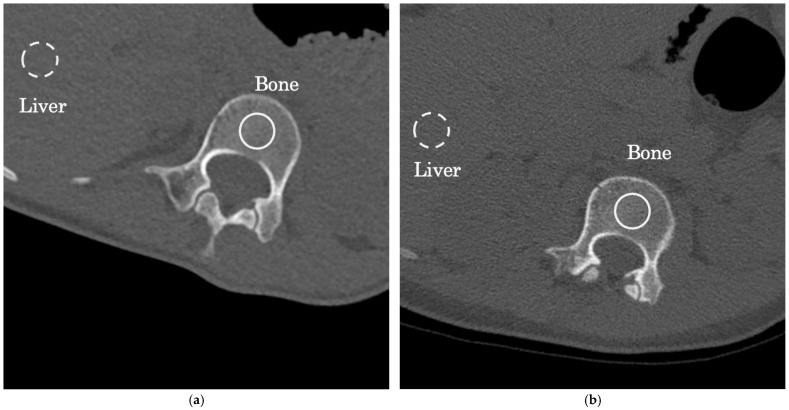
ROI (region of interest) setting points in the first lumbar vertebra (bone:) and liver for assessment of attenuation and image noise: (**a**) low-dose CT (LDCT); (**b**) normal-dose CT (NDCT).

The attenuation coefficient (in Hounsfield units, HU) and image noise (standard deviation of attenuation) were assessed for identical regions of interest (ROIs) within the first lumber vertebra and liver (Figure 2); the size of the ROIs was set to 5 mmφ for each ROI [39]. These parameters were employed to ascertain the signal-to-noise ratio (SNR) using the following formula [34]:

SNR = density_*Mean Attenuation of anatomic structure*_/noise_*Standard Deviation of HU of anatomic structure*_

### 2.6. Statistical Analysis

All statistical analyses were conducted using EZR (developed by Saitama Medical Center, Jichi Medical University, Saitama, Japan) [40]. The Shapiro–Wilk test was employed to determine the normality of the data distribution. Continuous variables were reported as mean ± standard deviation, and between-group comparisons were made using either an independent samples *t*-test for normally distributed data or a Mann–Whitney U test for non-normally distributed data. Pearson’s product—moment correlation coefficients were calculated to assess the relationships between dose parameters and physical indices. A *p*-value of <0.05 was considered statistically significant.

## 3. Results

### 3.1. Patient Characteristics

The demographic characteristics of the study patients in the two groups are summarized in Table 1. There were no statistically significant differences between the two groups in terms of age, sex, height, weight, or body mass index.

### 3.2. Radiation Dose

For CTDIvol, 0.76 mGy of LDCT and 2.94 mGy of NDCT, for DLP, 51.5 mGyx cm of LDCT and 207.1 Gy xcm of NDCT, and for effective dose ICRP publication 103, 1.1 mSv of LDCT and 4.8 mSv of NDCT were administered. In all cases, LDCT had lower radiation doses.

The equivalent doses were also lower for LDCT, such as 1.23 mSv of LDCT and 4.69 mSv of NDCT in the mammary gland, 1.15 mSv of LDCT and 4.53 mSv of NDCT in the uterus, and 1.06 mSv of LDCT and 4.27 mSv of NDCT in the ovaries. The total radiation dose applied in the LDCT group was significantly lower (by 75%) than in the NDCT group (*p* < 0.001). Data regarding the radiation dosage applied during both protocols are summarized in Table 2.

**Figure 3:**
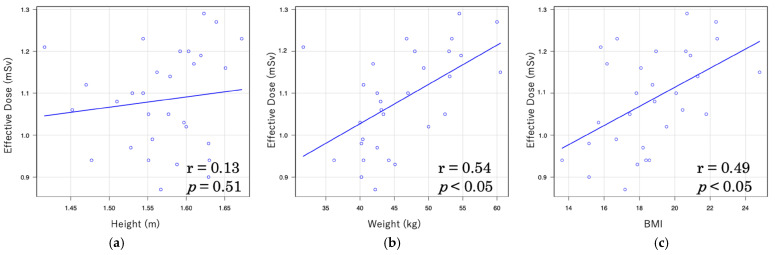
Relationship between effective dose and body indices in low-dose CT (LDCT): (**a**) Relationship of effective dose and height; (**b**) relationship of effective dose and weight; (**c**) relationship of effective dose and body mass index.

**Figure 4:**
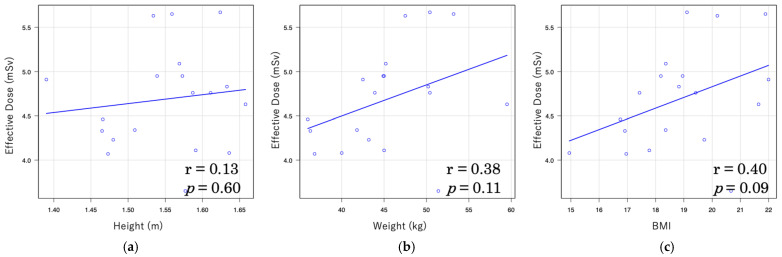
Relationship between effective dose and body indices in normal-dose CT (NDCT): (**a**) Relationship of effective dose and height; (**b**) relationship of effective dose and weight; (**c**) relationship of effective dose and body mass index.

In LDCT, the correlation between effective dose and body indices was moderately high, with coefficients of r = 0.54 for body weight and r = 0.49 for BMI (Figure 3). In contrast, the correlations in NDCT were weaker, with r = 0.38 for body weight and r = 0.40 for BMI (Figure 4).

**Figure 5:**
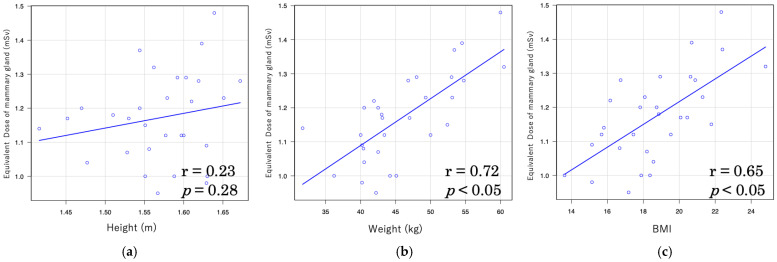
Relationship between equivalent dose of mammary gland and body indices in low-dose CT (LDCT): (**a**) Relationship of equivalent dose of mammary gland and height; (**b**) relationship of equivalent dose of mammary gland and weight; (**c**) relationship of equivalent dose of mammary gland and body mass index.

**Figure 6:**
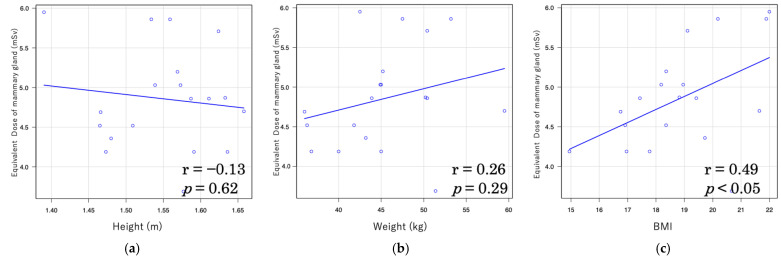
Relationship between equivalent dose of mammary gland and body indices in normal-dose CT (NDCT): (**a**) Relationship of equivalent dose of mammary gland and height; (**b**) relationship of equivalent dose of mammary gland and weight; (**c**) relationship of equivalent dose of mammary gland and body mass index.

Furthermore, the correlation coefficients between the equivalent dose to the mammary gland and body indices were relatively high in LDCT, with r = 0.72 for weight and r = 0.65 for BMI (Figure 5). In NDCT, however, the correlations were weaker, with r = 0.26 for weight and r = 0.49 for BMI (Figure 6).

Correlations between CTDIvol, DLP, and patient body size are presented in Table 3 and Table 4. In LDCT, CTDIvol showed strong correlations with body weight (r = 0.83) and BMI (r = 0.69), with the strongest correlation observed for body weight. Similarly, DLP showed moderate correlations with body weight (r = 0.78) and BMI (r = 0.57). In contrast, under NDCT conditions, only weak correlations were observed: CTDIvol with body weight (r = 0.33) and BMI (r = 0.38), and DLP with body weight (r = 0.48) and BMI (r = 0.33).

### 3.3. Image Quality Assessment

There was no statistically significant difference between the two protocols in terms of attenuation in the lumbar spine bone and liver tissue (*p* > 0.05; see Table 5). However, the image noise for both tissues was significantly higher in the NDCT group (*p* < 0.05; see Table 6). Furthermore, the SNR was significantly higher in the LDCT group for both lumbar spine bone and liver tissue (*p* < 0.05; see Table 7).

## 4. Discussion

We found that the use of LDCT with spectral shaping can decrease the overall radiation dose by 75% in comparison to NDCT for spinal CT imaging in AIS. In addition, LDCT was still effective, rather superior in image quality to NDCT, despite the dose reduction.

The effects of radiation from medical exposure are classified into deterministic and stochastic effects. Radiation effects are classified into deterministic and stochastic effects. Deterministic effects occur above a threshold dose and worsen with increasing exposure, leading to conditions such as skin erythema and cataracts due to cell death. In contrast, stochastic effects have no threshold, with their probability increasing with dose. These effects, including cancer and genetic mutations, result from DNA damage and are relevant even at low-dose exposure [41]. In angiography and interventional radiology, deterministic effects predominate, but in this study, stochastic effects predominate. Given that CT examinations are often repeated, the cumulative radiation dose may reach or exceed levels associated with increased cancer incidence in human populations [42].

A Danish follow-up study including AIS patients who received treatment with either bracing or surgery found that these patients were exposed to an average annual dose of 2.4–5.6 mSv and had an overall cancer rate of 4.3%, which was five times higher than that of normal subjects [22]. Another British registry study followed up patients who were exposed to CT radiation before the age of 22 years for the next 20 years and found that these patients have a 3.2 fold higher relative risk of leukemia and a 2.8 fold higher relative risk of brain tumors when exposed to 30 mGy or more of radiation, in comparison to those exposed to 5 mGy or less [43]. In adults, exposures below 100 mSv are safe and do not increase the relative risk of cancer [44]; however, a single exposure to CT in young children (who are exposed to an average of 4.5 mSv of radiation) increases the risk of childhood cancer development by 20% [45]. Furthermore, organ doses of 50–100 mGy, equivalent to several CT scans, may increase the risk of developing cancer [43,45,46,47].

Diana et al. reported that the effective dose in abdominal-pelvic CT was 14.8 mSv, with organ doses of 14.0 mGy for the mammary gland, 16.9 mGy for the uterus, and 19.6 mGy for the ovary [46]. In our study, the effective dose with LDCT using spectral shaping was only 1.1 mSv, and the organ doses were also significantly reduced, with 1.23 mSv for the mammary gland, 1.15 mSV for the uterus, and 1.06 mSv for the ovary. This approach can help significantly reduce the cancer risk for AIS patients who require repeated radiation exposure for monitoring. Therefore, medical staff must strive to curtail the effective radiation dose to which the children are exposed [48,49,50,51,52].

In this study, a moderate correlation was observed between effective dose and both body weight (r = 0.54) and BMI (r = 0.49) under LDCT conditions, whereas only a weak correlation was found under NDCT conditions (r = 0.38 and r = 0.40, respectively). Furthermore, under LDCT conditions, strong correlations were observed between organ doses of the mammary gland and both body weight (r = 0.72) and BMI (r = 0.65), whereas under NDCT conditions, only weak correlations were found (r = 0.26 and r = 0.49, respectively). A similar trend was noted for CTDIvol and DLP with patient body size: strong correlations were observed between CTDIvol and DLP and body weight under LDCT conditions (r = 0.83 and r = 0.78, respectively), and moderately strong correlations were found with BMI (r = 0.69 and r = 0.57). Under NDCT conditions, however, only weak correlations were observed with body weight (r = 0.33 and r = 0.48) and BMI (r = 0.38 and r = 0.33). These findings suggest that patient body size influences radiation dose in CT imaging. This trend is consistent with several previous studies reporting significant correlations between body size indices (weight, height, and BMI) and CT dose indices (CTDIvol and DLP), with body weight showing the strongest correlation [53]. Moreover, a study on liver CT demonstrated significant associations between anthropometric parameters and effective dose across all CT vendors, with effective dose increasing in accordance with height, weight, and BMI, in the order of weight > BMI > height [54]. These findings underscore the importance of adjusting radiation dose based on patient body size during CT examinations. In particular, individualized dose optimization is crucial for pediatric patients whose bodies are still developing and who are more sensitive to radiation exposure.

Several techniques are available to mitigate radiation exposure in CT; one of the most commonly used is reducing the tube voltage [27]. However, low tube voltage imaging necessitates a large tube current, which can lead to an insufficient dose and increased image noise [55]. Moreover, as the surface dose increases, there is a risk of mammary gland dose amplification, making low tube voltage imaging suboptimal [56]. The second technique is iterative reconstruction, which can significantly reduce image noise, improve overall SNR, and decrease the effective chest CT dose; nevertheless, increasing the intensity of iterative reconstruction may cause image blurring and texture changes [57,58]. Therefore, iterative reconstruction should be used judiciously, while accounting for image quality, when selecting the intensity.

The advent of the third-generation DSCT allowed the use of a spectral shaping technique employing an additional tin filter to optimize the X-ray spectrum. This technique eliminates low-energy photons having low-dose efficiency from the spectrum [59]. In comparison to 120 kV, a 100 kV with the tin filter produces lower doses at all depths, with only a slight change in dose at the phantom surface and center. This highlights the fact that the hardness of the line quality due to spectral shaping has greater energy transportation efficiency to the center than other voltages [60]. However, since the tin filter significantly reduces the intensity of the projected X-rays, a high current amplitude (in mAs) is essential to compensate for the photon loss. This places a considerable load on the X-ray tube, limiting the broad-scale use of the available equipment.

The third-generation iterative reconstruction algorithm, which combines statistical data modeling with model-based noise detection [37], can improve image quality and suppress the oil paint-like images observed in previous studies. Haubenreisser H et al. used non-contrast chest CT and reported a 90% dose reduction using a tin filter at 100 kV [34]. In comparison, we observed a 75% dose reduction with spectral shaping using a tin filter in the LDCT group, which may be due to the lower original radiation dose in the NDCT group, resulting in a relatively smaller difference.

The spectral shaping technique, which utilizes a tin filter to optimize the X-ray spectrum, is a promising imaging approach for CT imaging in children with AIS. This technique can potentially reduce radiation exposure while maintaining high image quality compared to conventional CT, making it a valuable tool in pediatric radiology. Besides the imaging benefits, this technique also allows for mitigating the risks associated with radiation exposure, particularly in children who may be more sensitive to its harmful effects. Therefore, it should be considered for routine use in the evaluation of AIS in children.

### 4.1. Limitation

The results of our study are limited by a lack of evaluation of the impact of this technique on the accuracy of navigation systems using CT images and the absence of comparison under identical conditions (using different CT systems). However, Noto et al. reported that the accuracy of the navigation system was not affected when using low-dose CT with spectrum shaping technology compared to conventional CT, and surgical outcomes were similar [61]. In this study, image quality was also comparable between LDCT and NDCT, suggesting no difference in navigation accuracy. According to the Radimetrics’ user guide, the dose management system uses CTDIvol and DLP recorded in the radiation dose structured report (RDSR) output from the CT equipment and performs a Monte Carlo simulation using a phantom applied from information, such as body thickness, and the patient’s age and sex, obtained from the CT images. Both equivalent and effective doses can be calculated by obtaining the average absorbed doses of organs and tissues and multiplying them by the radiation weighting and tissue weighting factors [62,63,64]. It has been reported that there is a 13% relative error between Monte Carlo simulation values and measurements using a physical phantom [65]. However, we did not verify the validity of these simulation results because radiation quality was not considered in this study. Finally, due to the limited sample size, we did not perform multivariable regression analyses adjusting for potential confounders such as BMI. Incorporating such analyses would further strengthen the validity of the findings, and this should be addressed in future larger-scale prospective studies.

### 4.2. Strengths and Future Directions

Despite these limitations, this study has several strengths. To our knowledge, this is the first study to evaluate spectral shaping CT with a tin filter specifically in AIS patients, with a comprehensive assessment of both effective dose and organ dose. The use of the Monte Carlo simulation-based dosimetry allowed reliable estimation of radiosensitive organ doses, such as the breast, uterus, and ovary.

Future research should include larger, multicenter prospective studies to confirm these results, incorporate quantitative image quality metrics, and evaluate the clinical impact on surgical planning navigation. Furthermore, individualized dose optimization strategies are especially important for pediatric patients, given their heightened radiosensitivity and cumulative exposure risks.

## 5. Conclusions

Spectral shaping with a tin filter combined with iterative reconstruction can reduce radiation dose by up to 75% compared to conventional methods. This technique can help mitigate the risk of radiation-induced cancers in children with AIS who require repeated imaging studies for monitoring purposes; therefore, it should be considered as an alternative approach to conventional CT in these patients.

## Figures and Tables

**Table 1:** Patient characteristics.

	LDCT * (*n* = 23)	NDCT * (*n* = 28)	*p* Value
Age (year)	15.3 ± 2.1	15.5 ± 1.7	0.923
Body length (cm)	157.0 ± 5.8	154.9 ± 5.6	0.185
Body weight (kg)	46.1 ± 6.1	44.3 ± 5.6	0.302
Body mass index (BMI)	18.6 ± 1.9	18.4 ± 1.8	0.636

* LDCT: low-dose CT, NDCT: normal-dose CT.

**Table 2:** Radiation dose.

	LDCT	NDCT	Reduction Rate	*p* Value
The volume CT dose index (CTDIvol) (mGy)	0.76 ± 0.09	2.94 ± 0.40	74.1%	<0.001
Dose Length Product (DLP) (mGy · cm)	51.5 ± 7.0	207.1 ± 33.8	75.1%	<0.001
Effective dose International Commission on Radiological Protection (ICRP) 103 (mSv)	1.1 ± 0.1	4.8 ± 0.7	77.1%	<0.001
Breast (mSv)	1.23 ± 0.12	4.87 ± 0.59	74.7%	<0.001
Uterus (mSv)	1.15 ± 0.11	4.66 ± 0.49	74.6%	<0.001
Ovary (mSv)	1.06 ± 0.10	4.45 ± 0.46	75.2%	<0.001

DCT: low-dose CT, NDCT: normal-dose CT.

**Table 3:** Correlation between height, weight, BMI, and CTDI.

	LDCT		NDCT	
	Correlation Coefficient (r) with CTDIvol	*p* Value	Correlation Coefficient (r) with CTDIvol	*p* Value
Height	0.371	<0.05	0.083	0.736
Weight	0.832	<0.001	0.333	0.164
BMI	0.694	<0.001	0.375	0.114

LDCT: low-dose CT, NDCT: normal-dose CT, CTDIvol: the volume CT dose index; BMI: Body mass index.

**Table 4:** Correlation between height, weight, BMI, and DLP.

	LDCT		NDCT	
	Correlation Coefficient (r) with DLP	*p* Value	Correlation Coefficient (r) with DLP	*p* Value
Height	0.489	<0.05	0.333	0.164
Weight	0.775	<0.001	0.477	<0.05
BMI	0.574	<0.001	0.33	0.167

LDCT: low-dose CT, NDCT: normal-dose CT, DLP: Dose length product; BMI: Body mass index.

**Table 5:** Mean attenuation (Hounsfield units, HU).

	LDCT	NDCT	*p* Value
Lumber spine	220.1 ± 24.9	235.7 ± 38.7	0.0941
Liver	63.08 ± 7.1	64.4 ± 3.7	0.65

LDCT: low-dose CT, NDCT: normal-dose CT.

**Table 6:** Image noise in anatomic regions of interest.

	LDCT	NDCT	*p* Value
Lumber spine	43.1 ± 5.4	70.6 ± 10.2	<0.05
Liver	37.6 ± 4.8	51.3 ± 6.8	<0.05

LDCT: low-dose CT, NDCT: normal-dose CT.

**Table 7:** Signal-to-noise ratio in anatomic regions of interest.

	LDCT	NDCT	*p* Value
Lumber spine	5.2 ± 0.9	3.4 ± 0.8	<0.05
Liver	1.7 ± 0.3	1.3 ± 0.2	<0.05

LDCT: low-dose CT, NDCT: normal-dose CT.

## Data Availability

The data presented in this study are available on request from the corresponding author upon reasonable request due to confidentiality concerns.

## Abbreviations

The following abbreviations are used in this manuscript:

AIS	Adolescent idiopathic scoliosis
BMI	Body mass index
CTDIvol	Volume computed tomography dose index
DLP	Dose length product
HU	Hounsfield unit
ICRP	International Commission on Radiological Protection
LDCT	Low-dose computed tomography
NDCT	Normal dose computed tomography
ROI	Region of interest
SNR	Signal-to-noise ratio
